# Special Issue: “Artificial Intelligence for Biomedical Signal Processing”

**DOI:** 10.3390/bioengineering12070753

**Published:** 2025-07-10

**Authors:** Fernando Vaquerizo-Villar, Verónica Barroso-García

**Affiliations:** 1 Biomedical Engineering Group, University of Valladolid, 47011 Valladolid, Spain; fernando.vaquerizo@uva.es; 2 Centro de Investigación Biomédica en Red en Bioingeniería, Biomateriales y Nanomedicina (CIBER-BBN), 47011 Valladolid, Spain

## 1. Introduction

Artificial intelligence (AI) has rapidly evolved from a conceptual promise into a practical and transformative asset in modern medicine [1,2]. Driven by significant advances in machine learning (ML), deep learning (DL), and biomedical signal and image processing, AI is reshaping the ways in which we understand, diagnose, and manage a wide range of clinical conditions [3,4]. This Special Issue of *Bioengineering* brings together a selection of innovative contributions that reflect the current state of the art in applying advanced AI-based computational techniques to solve real-world problems in healthcare.

The peer-reviewed studies cover a broad spectrum of applications, from contactless monitoring of physiological signals and predictive modeling in critical care settings to neural network-based approaches for neurorehabilitation [5,6,7,8,9,10]. They highlight how the integration of AI-based approaches, engineering, data science, and biomedical research is paving the way toward scalable, non-invasive, and clinically relevant solutions. These contributions not only demonstrate methodological rigor but also underscore a strong commitment to translational impact, with the potential to enhance clinical decision-making and improve patient outcomes [5,6,7,8,9,10]. Table 1 summarizes the peer-reviewed studies according to key criteria such as their biomedical signal, AI methodology, and clinical applications. A brief analysis of the featured papers is provided below.

## 2. Special Issue Articles

The Special Issue titled “Artificial Intelligence for Biomedical Signal Processing” contains six articles (as of 27 September 2024).

One of the central challenges in the analysis of physiological signals and images using AI lies in the quantity of available data. In this context, Li et al. (2024) introduced BioDiffusion [6], a generative diffusion model based on a modified U-Net architecture that is designed to synthesize biomedical signals. Although their study does not process signals directly, their model can generate realistic instances of physiological data such as electrocardiography (ECG) and accelerometry while preserving its key statistical, structural, and physiological properties. Its versatility enables the generation of synthetic data with high fidelity, positioning it as a strategic tool for synthetic dataset generation in digital medicine. This capability makes the approach particularly valuable for training and/or validating AI-based models when real clinical data are inaccessible, scarce, unlabeled, incomplete, and/or imbalanced.

Non-invasive vital signs monitoring is another major area of interest in applying AI algorithms to biomedical signals and images. In this context, the work by Cheng et al. (2023) proposed an AI-based approach to estimate blood oxygen saturation (SpO_2_) from facial videos [7]. Their approach encodes red–green–blue (RGB) facial videos into 2D spatial–temporal maps (STMaps) and estimates SpO_2_ levels from these maps using convolutional neural networks (CNNs), specifically ResNet-50, DenseNet-121, and EfficientNet-B3. The three CNN-based DL networks showed good performance, surpassing state-of-the-art methods and the international standard of 4% error for approved pulse oximeters. Sensitivity analyses also showed the influence of different color spaces, acquisition devices, and SpO_2_ ranges, while feature map visualization provided an identification of forehead, nose, and cheek facial features, which are relevant for SpO_2_ estimation [7]. This approach offers an accurate, rapid, and contactless alternative for SpO_2_ measurement that would be particularly beneficial in long-term monitoring scenarios requiring minimal physical contact, such as during infectious disease outbreaks or for patients with sensitive skin.

Similarly, Hwang et al. (2023) and Zhao et al. (2023) investigated the application of AI to the non-invasive monitorization of respiratory rate (RR) using biomedical signals [8,10]. Hwang et al. (2023) developed seven DL models, including a newly proposed dilated residual neural network, to estimate RR from photoplethysmography (PPG) data [8]. Their DL models showed promising results, although the authors acknowledged challenges such as motion artifacts and limited data diversity. Conversely, Zhao et al. (2023) proposed a transformer-based model, called TransRR, to predict RR from PPG and ECG data [10]. TransRR outperformed conventional CNN-based and long short-term memory (LSTM)-based models, although the authors acknowledged that more data are required to train a highly generalizable and clinically reliable RR estimation model. Overall, Hwang et al. (2023) and Zhao et al. (2023) highlight the potential of AI-based approaches in enhancing non-invasive RR monitoring across diverse clinical settings, including ICUs, emergency departments, and intensive care patients [8,10].

Extending the application of AI in non-invasive monitoring to ventilatory management, Park et al. (2023) evaluated a CNN-based DL model to predict weaning outcomes from ventilatory parameters that were obtained from ventilators during spontaneous breathing trials (SBTs) [9]. Particularly, a multi-modal CNN was developed for weaning prediction using pressure, flow, and volume time waveforms, as well as twenty-five numerical features from each breath. The model demonstrated strong predictive performance, with superior results compared with the conventional rapid shallow breathing index (RSBI) method. Park et al. (2023) also offered visual explanations of the waveform features that influence the predictions of the CNN using gradient-weighted class activation mapping (Grad-CAM) [9]. By relying solely on ventilator-derived data, this AI-driven approach provides a non-invasive method for assessing a patient’s readiness for weaning, highlighting its potential to enhance patient care and optimize ventilator management in critical care settings.

Finally, the study by Kaviri and Vinjamuri (2024) focused on the analysis of EEG signals to support neurological rehabilitation processes by means of motor imagery following a stroke [5]. Their approach combines EEG source localization (ESL), a technique that transforms multichannel EEG signals into three-dimensional representations of cortical activity, with a residual convolutional neural network (ResNetCNN). The proposed ResNetCNN architecture incorporates residual convolutional blocks with shortcut connections, which facilitate deep network training and prevent gradient degradation in architectures with many layers. This design enables the capture of complex spatial patterns of cortical activity from EEG recordings while maintaining training stability and depth. As a result, their method not only improves the accuracy of identifying post-stroke active brain regions but also offers a robust architecture for functional brain activity mapping [5]. This is particularly valuable for optimizing patient monitoring and planning personalized rehabilitation therapies based on cortical activation patterns.

Thus, beyond the specific methodologies and types of signals employed, it is essential to examine the technical core of each study, where AI is thoughtfully tailored to address concrete biomedical problems through well-founded approaches that are clearly oriented toward clinical practice.

## 3. Conclusions

The six articles featured in this Special Issue of *Bioengineering* serve as compelling evidence of the transformative potential of AI in biomedical signal processing. From patient monitoring and clinical outcome prediction to the generation of synthetic data, these contributions offer a glimpse into the future of real-world clinical settings and digital medicine. These AI-based approaches are paving the way toward more precise, accessible, and human-centered healthcare.

We encourage the scientific community to explore these works in depth and continue fostering collaborative research that can help translate these technologies from the laboratory to the patient’s bedside.

## Figures and Tables

**Table 1:** Comparative overview of the articles published in the Special Issue “Artificial Intelligence for Biomedical Signal Processing”.

No.	Reference	Biomedical Signal	AI Methodology	Clinical Application
1	Kaviri et al., 2024 [5]	EEG	Source localization + residual CNN	Motor imagery in post-stroke rehabilitation
2	Li et al., 2024 [6]	Multimodal (synthetic, accelerometer, and ECG signals)	Diffusion generative model	Biomedical signal synthesis
3	Chen et al., 2024 [7]	Facial videos (RGB)	CNN (ResNet-50, DenseNet-121, EfficientNet-B3) + feature map visualization	Remote SpO_2_ measurement and identification of relevant facial features
4	Hwang et al., 2023 [8]	PPG	Dilated residual neural network	Continuous respiratory rate estimation
5	Park et al., 2023 [9]	Numerical and waveform ventilatory parameters	Multimodal CNN + Grad-CAM	Weaning outcome prediction and relevant feature identification
6	Zhao et al., 2023 [10]	ECG + PPG	Transformer	Respiratory rate estimation

AI = artificial intelligence; CNN = convolutional neural network; ECG = electrocardiogram; EEG = electroencephalogram; Grad-CAM = gradient-weighted class activation mapping; PPG = photoplethysmography; RGB = red–green–blue; SpO_2_ = oxygen saturation.

## Abbreviations

The following abbreviations are used in this manuscript:

AI	Artificial intelligence
CNN	Convolutional neural network
DL	Deep learning
ECG	Electrocardiogram
EEG	Electroencephalogram
ESL	EEG source localization
Grad-CAM	Gradient-weighted class activation mapping
LSTM	Long short-term memory
ML	Machine learning
PPG	Photoplethysmography
ResNetCNN	Residual convolutional neural network
RGB	Red–green–blue
RR	Respiratory rate
RSBI	Rapid shallow breathing index
SBTs	Spontaneous breathing trials
SpO_2_	Oxygen saturation
STMaps	Spatial–temporal maps
